# Microbial community shifts induced by plastic and zinc as substitutes of tire abrasion

**DOI:** 10.1038/s41598-022-22906-6

**Published:** 2022-11-04

**Authors:** G. Sieber, D. Beisser, J. L. Rothenberger, M. Shah, M. Schumann, B. Sures, J. Boenigk

**Affiliations:** 1 Biodiversity, University of Duisburg-Essen, Essen, Germany; 2 Centre for Water and Environmental Research, University of Duisburg-Essen, Essen, Germany; 3 Aquatic Ecology, University of Duisburg-Essen, Essen, Germany; 4 Research Center One Health Ruhr, University Alliance Ruhr, Environmental Metagenomics, University of Duisburg-Essen, Essen, Germany

**Keywords:** Biological techniques, Computational biology and bioinformatics, Ecology, Microbiology

## Abstract

Aquatic environments serve as a sink for anthropogenic discharges. A significant part of the discharge is tire wear, which is increasingly being released into the environment, causing environmental disasters due to their longevity and the large number of pollutants they contain. Main components of tires are plastic and zinc, which therefore can be used as substitutes for tire abrasion to study the effect on microbial life. We investigate environmentally realistic concentrations of plastic and zinc on a freshwater microeukaryotic community using high-throughput sequencing of the 18S V9 region over a 14-day exposure period. Apart from a generally unchanged diversity upon exposure to zinc and nanoplastics, a change in community structure due to zinc is evident, but not due to nanoplastics. Evidently, nanoplastic particles hardly affect the community, but zinc exposure results in drastic functional abundance shifts concerning the trophic mode. Phototrophic microorganisms were almost completely diminished initially, but photosynthesis recovered. However, the dominant taxa performing photosynthesis changed from bacillariophytes to chlorophytes. While phototrophic organisms are decreasing in the presence of zinc, the mixotrophic fraction initially benefitted and the heterotrophic fraction were benefitting throughout the exposure period. In contrast to lasting changes in taxon composition, the functional community composition is initially strongly imbalanced after application of zinc but returns to the original state.

## Introduction

**Figure 1:**
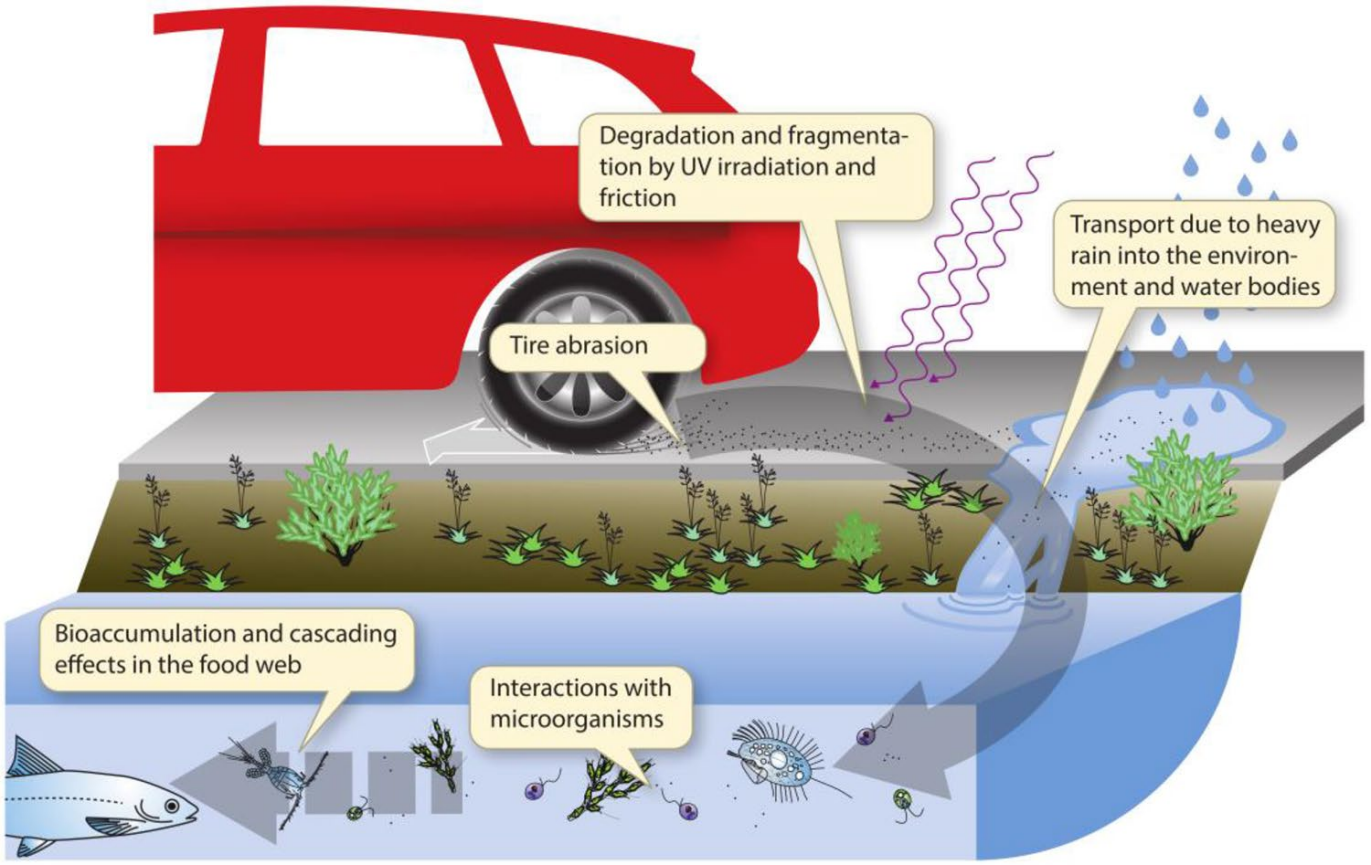
Pictorial representation showing how pollutants from tire abrasion enter aquatic ecosystems. While driving, the tire wears off and leaves particles on the road surface, which are further degraded to micro-and nanoparticles by friction and UV radiation. These particles are discharged with the next heavy rainfall into aquatic and terrestrial ecosystems. There they interact and affect microbial organisms and are further accumulated along the food chain^17^.

Pollution is a globally growing problem, triggering unforeseen consequences and incalculable harm to biodiversity^1–6^. One important pollution source is tire abrasion^7,8^. Tires contain numerous substances like carbon black, additives, fabric, curing agents, sulfur, metals, but also styrene and butadiene, which are precursors of polystyrene^9–12^. Wearing off the tire leaves particles on the surface, which, due to abrasion and UV radiation, degrade to micro- and nanoplastic^4,13–16^. During the next heavy rainfall event, these particles are then carried into the water bodies (Fig. 1).

The particles can remain in the environment for centuries, if not millennia, due to their long half-life, attributable to their low biodegradability^18^. One of the most important toxic compounds in tires in terms of quantity is zinc, i.e., tires contain around 1% zinc^19^. Some adverse effects are already described^20–24^, but the overall impact on microeukaryotic communities are insufficiently investigated yet^5,25–29^ as the effects of nano- and microparticles and associated toxic substances are mostly unknown^30^.

Further, for tire abrasion it is unclear to what extent potential effects are caused by the nanoplastics or by toxic compounds adsorbed to or embedded in the nanoplastic particles^4,7,8,13,28,31,32^.

Strong effects on microeukaryotic communities are likely as both direct and indirect harmful effects via physical contact, phagocytosis, feeding and accumulation via the food web are likely^37–40^. A shift in bacterial communities has been shown by Fu et al. suggesting indirect effects on microeukaryotes i.e. through feeding interactions^41,42^. Effects of micro- and nanoplastics are known to increase with decreasing particle size^43–47^. Nanoplastics can permeate into lipid membranes^48^ leading to lipid peroxidation^49^ and to cell membrane disruption by direct contact, physical piercing, plasmolysis and physiological stress^20,50–54^. Nano- and microplastics have further been shown to cause a decrease in chlorophyll content and photosynthetic activity in microalgal cultures, independent of growth inhibition and shading effects^43,52,55,56^. The underlying effects are diverse, ranging from reduced CO_2_ uptake to increased reactive oxygen species (ROS) production, distorted thylakoids and negatively affected photosynthesis genes^43,52,55,56^.

Zinc, on the other hand, is one of the most common and abundant metals in the environment^57^. Even though essential for cells at low concentration, it is toxic at higher concentrations due to several effects on the physiology of cells^58,59^. For instance, excess zinc can lead to lipid peroxidation, decreasing cellular chlorophyll content, photosynthesis reduction and reduced diversity in microorganisms^59–61^. Zinc excess can further negatively affect the activities of carbon-, nitrogen-, and phosphorus-acquiring enzymes^62^. In order to cope with zinc individual microeukaryotes activate defense mechanisms such as the production of antioxidants and metal chelators^63^.

In this study, we examined the effects of 100 nm polystyrene nanoparticles and zinc as a substitute for tire abrasion, and its effects on a natural freshwater microbial community using mesocosms in conjunction with next generation amplicon sequencing.

The objectives of this study were to determine effects on the community composition both for the polystyrene nanoparticles and for zinc. Regarding nanoplastic we particularly hypothesize a decrease of microeukaryotes lacking cell walls or possessing (thin) cellulose but hardly an effect on taxa with robust cell walls such as diatoms and fungi and thus an increase of the relative abundance of the latter. Nanoplastics may invade the cells of the former taxa more easily, damage the cellulose wall and thylakoid membranes, while the latter taxa should be better protected from such effects^52^. We conjecture effects on phototrophs and heterotrophs, however we assume that heterotrophic microeukaryotes are affected more severely due to weaker defenses, mistaking plastic for food and accumulation compared to phototrophic microeukaryotes and fungi which should be reflected by a relative decrease of phagotrophs in the microbial community. Nevertheless, negative effects on photosynthesis have also been shown for cultivated microorganisms^52,56^. Such effects may decrease the abundance of phototrophic microorganisms and counterbalance the expected community shifts.

With respect to zinc, we expect to see a greater impact onphototrophic microorganisms than on heterotrophic or mixotrophic ones as it impacts nutrient acquiring enzymes and negatively affects photosynthesis. The impact of zinc on cells, however, may be modulated by interactions with outer structures such as cell walls. Biomolecules present in cell walls such as mannans, glucans, phosphomannans, chitin, chitosan and melanin are known to act as bioabsorbants of metals^64^. We therefore expect that organisms possessing thick and robust cell walls, in particular fungi, are more tolerant to zinc and that consequently their relative abundance will increase^65^. Thinking along these lines, we hypothesize strong negative effects for phagotrophic taxa as most of these taxa lack cell walls and further accumulate zinc along the food chain. In consequence, we further hypothesize a decrease of heterotrophs, an increase of fungi and a presumably intermediate response of phototrophic taxa. However, as metal binding to cell walls should eventually reduce the free zinc concentration, the mid- to long-term effects may be the other way around and organisms lacking zinc-binding molecules on the surface may benefit.

## Methods

Lake water was taken from lake Baldeney (Germany, Essen 51.402484 N, 7.007840 E) on the 22 nd of August 2019 5 m from the shoreline. Water was filtered through a stainless steel filter (2 mm) and subsequently through a 100 μm mesh to exclude larger metazoa and debris. The water was stored indoors in an open 2400 L stainless steel tank. To compensate for missing substrate we added an amount of 0.025% of NSY medium (Nutrient broth, peptone from soybean and yeast extract media) and WC medium (freshwater medium) (modified from Guillard & Lorenzen^66^) (Table S1) every day. The water was exposed to daylight, gently stirred for 30 s once a day and ventilated by four aquarium aerators. After two days of initial acclimatization, we transferred the water into 25 glass aquariums (45 L each) with one aquarium aerator each and allowed for a secondary acclimatization for one day. The experiment was run under natural light conditions in an air-conditioned greenhouse at 19 °C air temperature. Basic chemical parameters, such as pH, conductivity and water temperature, are shown in the supplement (Table S2).

In the experiment we tested the effects of polystyrene nanoparticles (Micromod, Germany, 100 nm polystyrene, micromer®-F green) and zinc (applied as zinc chloride) in a replicated factorial design^57,67^. In addition, we ran acontrol containing silicate nanoparticles (Micromod, Germany, 100 nm silica, sicastar®-F). We applied nanoparticles in a concentration of 3 × 10^8^ particles ml^−1^ and zinc chloride in a concentration corresponding to 1 mg Zn l^−1^ (15.3 µmol/l). The experimental setup therefore consisted of five different treatments with five replicates each. Namely, control (without any exposure), zinc exposure (Zn), nanoplastic exposure (NP), zinc & nanoplastic (ZnNP), and the additional silica nanoparticle exposure (Si). The silica nanoparticle treatment was applied to differentiate between effects due to particle concentration from effects due to particle composition (polystyrene and silica). The nanoplastic concentration (0.158 μg/l) applied corresponds to concentrations found in the environment^68^. Baselines of metals and actual zinc concentrations are shown in the supplement (Tables S3, S4). Dissolved metals were measured by measuring filtered samples (using Whatmann™ 0.45 μm cellulose nitrate filters). The metals concentrations were analyzed using inductively coupled plasma mass spectrometry (ICP-MS). The analyses were carried out with a quadrupole ICP-MS system (Perkin Elmer Sciex Elan DRC-e) operating at 1000 W plasma power, 14 l/min plasma gas flow and 0.95 l/min nebuliser gas flow and an auto sampler system (Perkin Elmer AS-90) connected with a peristaltic pump with a sample flow of 1 ml/min. To avoid contamination and memory effects the wash time between measurements was set to 10 s (with 1% HNO_3_, suprapure). Before analyses, the samples were diluted 1:10 using a solution of 1% HNO_3_ (suprapure) with a concentration of 10 ng/l of yttrium (Y) as internal standard. In order to control the accuracy and stability during measurements a standard solution of all analytes in concentration of 10 µg/l was analyzed after every 10 samples. The calibration was carried out with a series of 1 1 dilutions of a multielement standard solution (ICP Multielementstandard solution, Merck, Darmstadt, Germany) using additionally Molybdenum standard (AAS-standard, Bernd Kraft, Duisburg, Germany). Element concentrations were calculated as mg l^-1^ using corresponding regression lines (correlation factor ≥ 0.999)^69,70^. A lower dissolved zinc concentration than calculated is present under natural conditions, as zinc is bound to organisms and particles and is therefore not freely available (Fig. S1). Pre-experiments confirmed that no zinc adsorption to the aquariums was detectable. Formation of nanoplastic aggregates, however, occurs naturally and cannot be overcome without addition of surfactants to ensure initial dispersion, which is not feasible in the experiment; thus particles were sonicated in 0.1 M Na_2_CO_3_ for 15 min^71,72^ (Manufacturer) prior to the experiment. Corresponding amounts of Na_2_CO_3_ were added to the treatments without nanoparticles to exclude effects caused by Na_2_CO_3_.

### Sample processing

Samples were taken after 1 h, 12 h, 24 h, 48 h/2 days, 96 h/4 days, 168 h/7 day and 336 h/14 days after stirring and before addition of nutrient media. This sampling scheme was chosen to capture immediate effects at the beginning with a dense sampling and medium-term effects, which become rather visible only after a few generations, with a more broader sampling towards the end. Briefly, we withdrew 400 ml (sampling points 1, 12 and 24 h) and 200 ml (sampling points 48, 96, 168, 336 h) with sterile 1 L glass bottles and filtered the sample on a polycarbonate filter (0.2 µm). Sampling volume was less for the last four samplings as filters clogged earlier.Saturation curves were used to manually rule out that samples were under-sampled. This sampling scheme is considered biomass normalized because filters that are close to clogging were sampled, so that sampled biomass is comparable.

From each sample, 4 ml subsamples were taken and adapted to darkness (30 min) for measuring the photosynthetic activity using the AquaPen-C AP100 QJIP protocol (Photon System Instruments, Brno). Each subsample was measurement twice (2 times 2 ml) and the average was taken for analysis (Table S5).The Fv/Fm quotient describes the maximum photochemical quantum yield of the photosystem II (PSII) in a dark adapted state, with Fv being the variable fluorescence or difference between maximum and minimum yields (Fm-F_0_), of chlorophyll a fluorescence. The measured `Fv/Fm`- ratio is related to the photochemical conversion efficiency of the PSII and describes the proportion of functional PSII indicating stress and vitality. The `Fix Area` value measures the area under the induction curve of fluorescence during a saturation light flash (1 s), which serves as an indirect proxy for the chlorophyll a content^73–75^. Photosynthesis proxies were statistically compared against the control using Wilcoxon tests.

Filters were air dried and immediately stored in 1.5 ml eppendorf tubes with 400 μl RNA/DNA Shield (Zymo Research) and frozen at -80 C° until DNA extraction. DNA was extracted using the Zymo Quick DNA/RNA microprep plus kit with a modified protocol. Briefly, each step was performed at room temperature and samples were centrifuged until the fluid passed the columns (30–70 s) at 10.000 rpm.Two filters with the surrounding DNA/RNA shield solution were transferred in a bashing bead tube (Zymo BashingBeads Lysis Tubes (0.1 & 0.5 mm)) and homogenized using the FastPrep instrument (MP Biomedical). Homogenization was run five times for 30 s at 5.5 m/s. Between homogenization steps the samples rested 1 min on ice. The bashing bead tubes were centrifuged for 30 s, the supernatant was transferred in a 1.5 ml tube and mixed with 400 μl lysis buffer. After 30 s the sample was transferred onto a Zymo-Spin IC-XM column in a 1.5 ml RNAse free tube and was centrifuged.

400 μl preperation buffer was added to the DNA carrying column and the column was centrifuged. Followed by two washing steps with 700 and 400 μl wash buffer and a final dry centrifugation for 2 min. The dry columns were transferred to a 1.5 ml tube and 30 μl RNAse/DNAse free water was added. After 5 min an elution centrifugation was performed. DNA was frozen at − 20 °C.

PCR amplification targeted the 18S V9 region. Forward primers are based on 1391f. (5`-GTACACACCGCCCGTC-3`)^76^. Reverse primers are based on EukR (5`-TGATCCTTCYGCAGGTTCACCTAC-3`)^77^. The final concentrations in all of the PCR reactions were as follows: 0.5 μl of DNA template in 25 μl PCR reactions with 0.02 units/μl of Q5 DNA polymerase (NEB), 5 μl Q5 buffer, primers at a final 0.5 μm concentration, dNTPs at 0.2 μm final concentration and 16.25 μl water. The PCRs conditions included an initial denaturation at 98 °C for 30 s and 25 cycles of: 98 °C for 30 s, annealing for 10 s at 60 °C, extension for 15 s at 72 °C, followed by a final extension step at 72 °C for 2 min.

DNA-Sequencing: Equimolar subsamples were pooled and commercially sequenced using a NovaSeq6000, yielding 150 bp-long paired-end reads (Fasteris, Geneva, CH).

### Bioinformatics

Adapter removal, quality trimming and demultiplexing using molecular identifier (MID) sequences were performed by the sequencing company (Fasteris). The base quality of the sequence reads was checked using FastQC^78^. A split-sample filtering protocol for Illumina amplicon sequencing was used for two technical replicates per DNA sample^79^. The raw sequences were quality filtered^80^ (PRINSEQ-lite v.0.20.4) to remove reads with an average Phred quality score below 25. The paired-end reads were assembled and quality filtered with PANDASeq^81^ (v2.10). All reads with uncalled bases, an assembly quality score below 0.9, a read overlap below 20, or a base with a recalculated Phread-score below 1 were removed. After dereplicating chimeras were identified and filtered using UCHIME^82^ (v7.0.1090) with default settings. Sequences that were not present in both sample branches were discarded^79^. Operational taxonomic units (OTUs) were created swarm with default settings^83^ and amplicon sequencing variants (ASVs) were created with DADA2^84^ (1.18.0). Sequences can barely be tracked down to represent distinct species or even strains, thus, the use of taxonomic units (OTUs or ASVs) is mandatory to perform analyses. The tools were used within the modular bioinformatic pipeline Natrix^85^. Sequences wereBLAST (Basic Local Alignment Search Tool) aligned against the National Center for Biotechnology Information (NCBI) nucleotide database (nt) (downloaded Feb. 2020). OTUs with less than 100 reads in total, occurring in less than 10 samples, having a BLAST e-value larger than 1 × 10^–5^ or a percentage of identity less than 90% were discarded. Sequencing depth waschecked with rarefaction curves and samples that did not reach saturation were manually curated. Namely, the samples NP1_336h, NP5_168h, ZnNP5_168h, Zn5_336h, C2_336h were removed before analysis.

True diversities and functional group abundances were computed from rarefied reads (function rrarefy from the vegan R Package^86^, version 2.5.7^87^). Reads were rarefied to the mean read amount (419,158) from all samples. True diversities are based on the Simpson index using the R package RAM^88^ as well as Pielou`s evenness. True diversity, also known as Hill numbers or effective number of species, were chosen as a measurement for diversity as it is not a non-linear diversity index (e.g. Shannon index, Simpson index) but is suitable for linear comparisons between samples. True diversity and OTU richness was statistically compared against the control using Wilcoxon tests.

We assigned the nutritional mode to OTUs by using their BLAST based percentage identity values to species/taxa where the nutritional mode is known. We accepted individual thresholds for correctly resolving the assigned taxonomy to the phylum, clade, class, order, genus, species and strain level, while referring to Table S6. For the phylum/clade/class/order level we accepted a percentage identity of 90%, for the family level 92%, for the genus level 94.5%, for the species level 98.7% and for the strain level 99%^89^. These thresholds were derived from research on bacteria and archaea and were used here for eukaryotes, since similar, universally usable, thresholds are not available for the V9 18S region in eukaryotic microorganisms. Differential abundant OTUs were calculated by their log_2_fold changes using the R Package DESeq2^90^ on raw count data with an accepted adjusted *p* value below 0.01. The design formula accounts for effects caused by individual treatments and not by temporal effects (design = ~ time + treatment).

For the non-metric multidimensional scaling (NMDS) we replaced zeroes in our dataset based on a Bayesian-multiplicative replacement (cmultRepl, R zCompositions package^91^) and calculated the Aitchison distance, as we are dealing with compositional data^92^. Aitchison distance is used as a community dissimilarity indicator. This distance matrix was used to compare the treatments among each other using a Benjamini–Hochberg p-adjusted pairwise adonis (adonis padj). The same test wasapplied to functional community compositions of individual treatments. Figures were created with R and Adobe Illustrator^17,87^.

### Data availability

The datasets generated and analyzed during the current study are available in the Genbank repository (PRJNA844210) available after the manuscript was accepted.

## Results

### Sequencing results

The total number of assembled reads after filtering was 70,807,761 which formed 1410 OTUs. Of these 419,158 ± 182,342 assembled reads and 571 ± 189 OTUs were found in individual samples. Results shown here are based on OTUs as patterns were inconclusive for ASVs (Fig. S2).

### Effects of treatments on the community structure

**Figure 2:**
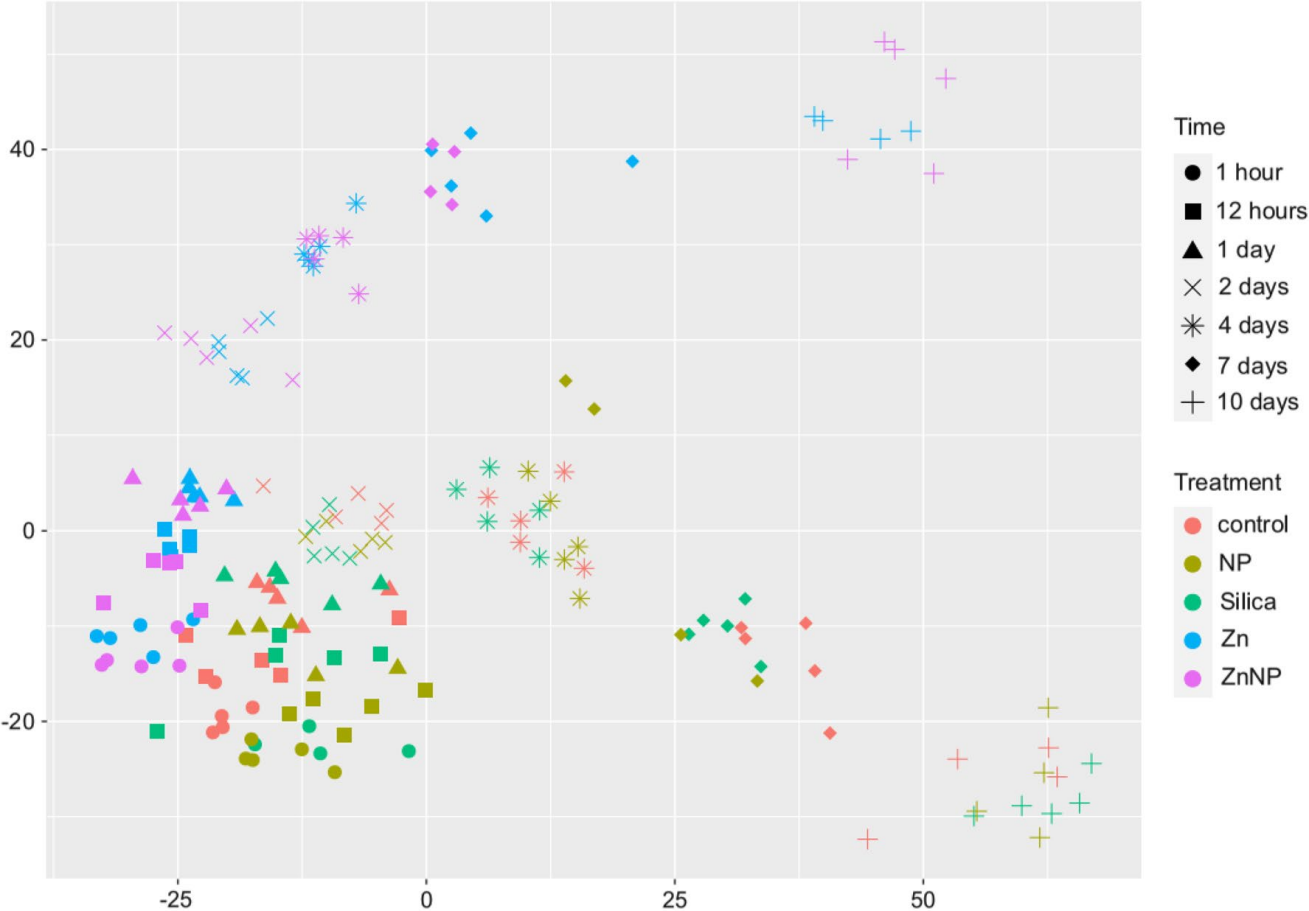
Non-metric multidimensional scaling (NMDS) ordination plot of individual treatments over time showing their community development and differentiation during the time-course of the experiment. A stress value of 0.105 denotes a decent ordination representation. Treatments are color-coded and sampling points are shape-coded. It can be observed that the x-axis represents time and the y-axis individual treatments Samples close to each other are more similar, thus their community structure is more similar than the community structure of more distant samples. The distance between individual samples is rank-based and not linear. The differentiation of the Zn and ZnNP treatment compared to the control becomes obvious over time, while there is no differentiation of Si and NP treatment, when compared to the control.

Microeukaryotic communities in the different treatments differed and the separation increased over time. Besides a general temporal development we observed a pronounced effect of zinc, i.e. the shift of the community composition was stronger in treatments exposed to zinc while exposure to silicate and polystyrene nanoparticles did not or hardly affect the development of the community compared to the control treatment (Fig. 2). The community turnover and the impact of zinc became visible both on the level of individual OTUs (Fig. 2) as well as on the level of higher-level groups (S3).

**Figure 3:**
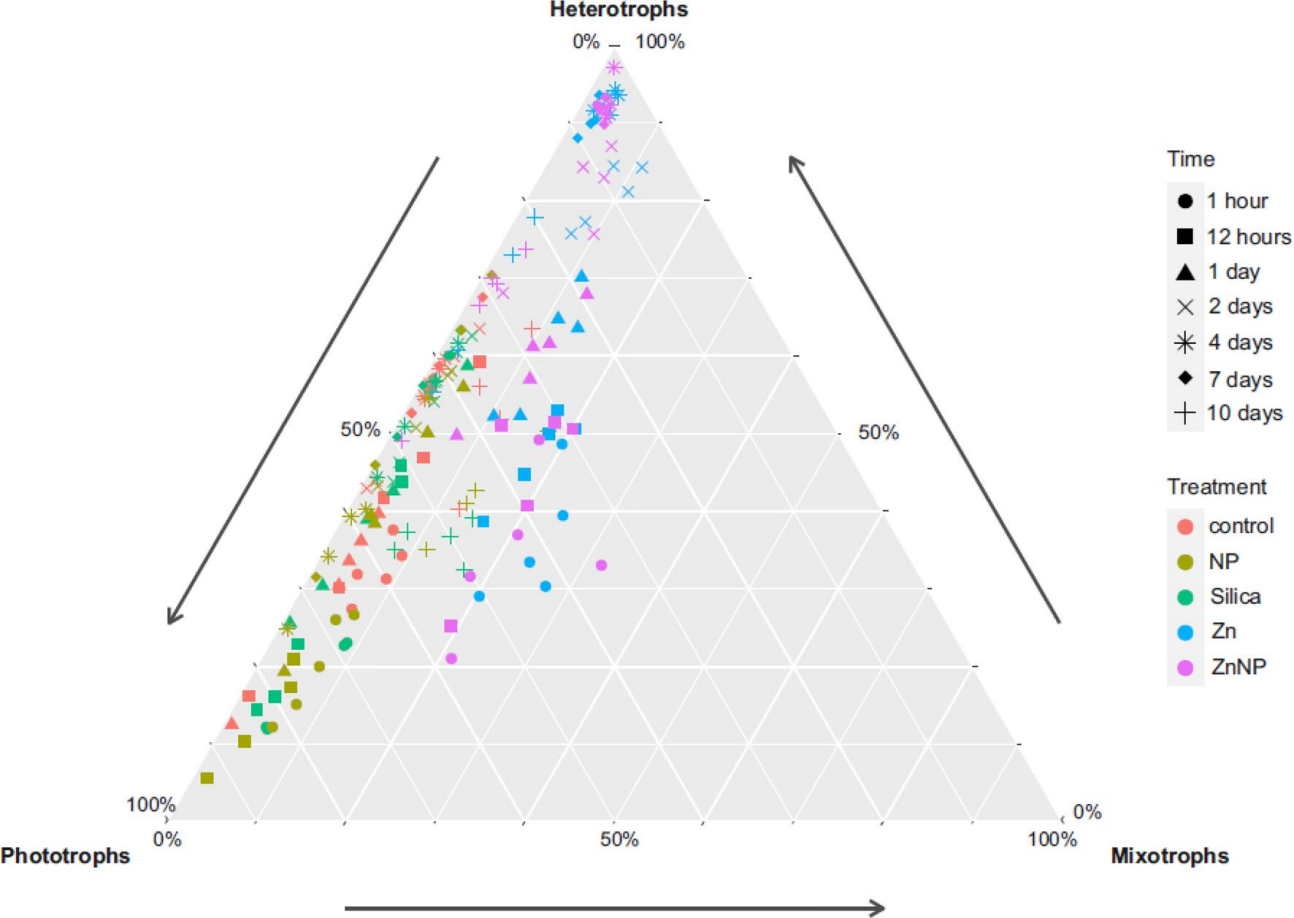
Ternary plot showing the functional community composition and its shift. Treatments are color-coded and sampling points are shape coded. Consumers include parasitic and fungal OTUs. Ternary plots are three dimensional composition plots. Relative abundances of samples always sum up to 100%. The closer a sample is located towards one corner (nutrition type), the more organisms affiliated with this nutritional type are represented in the community in terms of relative abundance. Samples close to each other are similar in their functional community composition. Distances are based on the relative abundance of the three functional groups, thus distances between samples behave linear.

While community composition developed similarly within treatments without additional Zn (control, silicate and NP) as well as within zinc-treatments (Zn and ZnNP) the development was different between these two groups (pairwise adonis padj > 0.01; Fig. 2). The communities in the zinc treatments started diverging from the zinc-free treatments already within the first hour after exposure (Figs. 2, 3) and continued to diverge until the end of the experiment. In particular, the relative abundances of Chrysophyta and Euglenozoa were initially higher in the zinc-treatments but later replaced by a higher abundance of Choanoflagellata, Cercozoa, Amoeba and Chlorophyta. While there are generally less Bacillariophyta, Chytridiomycota and Dinophyta (Fig. S3) compared to zinc-free treatments.

The exposure of silica and polystyrene nanoparticles did not significantly affect the development of the community. Likewise, we did not find indications for an interactive effect of nanoplastic and zinc on the community structure.

### Diversity

While taxon composition was changing during the course of the experiment and developed differently between zinc and non-zinc treatments (Fig. 2) the diversity was hardly affected: OTU richness did not significantly change in the course of the experiment (padj > 0.01) (Fig. S4, Table S8). Similarly, the true diversity, as well as evenness, was not significantly affected throughout the experiment (wilcoxon *p* > 0.01) (Figs. S5, S6). Further, diversity indices were similar between zinc and non-zinc treatments (wilcoxon *p* > 0.01). Diversity is thus decoupled from the taxonomic community profile. Although the relative abundances of individual taxa/OTUs changed, the evenness did not.

### Functional attributes

Roughly 90% of the OTUs (1273 OTUs, i.e. 702 heterotrophic, 26 mixotrophic, 41 parasitic, 504 phototrophic) were appointable to a trophic mode, i.e. phototrophic, mixotrophic, heterotrophic or parasitic, while 137 OTUs could not be assigned to a trophic mode.

The relative importance of trophic modes changed over time and the strength of this change depended on the treatment, i.e. the shift was stronger in the zinc treatments as in the non-zinc treatments. Within groups, i.e. between control, Si and NP on the one hand and between Zn and ZnNP on the other, no significant differences were observed (pairwise adonis, *p* > 0.01), while the difference between these groups is significant (pairwise adonis, *p* < 0.01).

Initially phototrophs dominated in all treatments. During the course of the experiment, the community shifted towards a rather heterotrophic dominated community and subsequently back to a more balanced community with a slight dominance of heterotrophs. In the non-zinc treatment the shift of nutritional modes was moderate while in the zinc-treatments the initial shift was sharp and phototrophs decreased drastically. In consequence, the relative importance of mixotrophs increased initially in the zinc treatment and later on the community was nearly exclusively composed of heterotrophs. Towards the end of the experiment, phototrophs became more frequent again and functional diversity became similar to the control in the zinc treatment as well. Thus, the ratio of consumers, mixotrophs and phototrophs is largely balanced towards the end and became similar to the starting conditions in all experiment. However, taxon composition changed strongly (Tables S8, S9, S10, S11).

In the zinc treatments (Zn and ZnNP) the dominant phototrophs of the initial community, i.e., Bacillariophyta, were nearly entirely replaced by Archaeplastida as the experiment progressed (Fig. S10). It is noteworthy that the Chlorophyta species *Halochlorella rubescens* made up a major part of the phototrophic fraction. Considering the last sampling day this OTU made up 16.8 ± 9.9% of the total reads in the Zn and 13.3 ± 9.1% in the ZnNP treatment, representing 63.5 ± 31.8% and 40% ± 14.2 of the Chlorophyta fraction, respectively.

The mixotrophic subcommunity is diminished in the zinc treatments (Zn and ZnNP) and consisted mostly of Chrysophyta with a small share of Dinophyta. In contrast, Dinophyta dominated this fraction almost exclusively in the non-zinc treatments (control, Si and NP) (Table S10).

In all treatments heterotrophs were initially dominated by Chrysophyta and Ciliophora while they were Cercozoa and Ciliophora dominated towards the end of the experiment. However, their relative importance differed, as the zinc treatments contained a higher fraction of Amoebozoa and Cercozoa but a lower fraction of Ciliophora and Chytridiomycota. Interestingly, the NP treatment contained more Cercozoa than any other treatment (Table S11) which is one of the few deviations of the NP treatment from the control.

The relative abundance of parasites decreased in the zinc treatments and that of fungi increased in the non-zinc treatments towards the end of the experiment.

### Fv/Fm photosynthesis proxy

The shift of trophic modes is supported by photosynthesis proxies (Fig. S7). While photosynthesis proxies do not significantly vary in the non-zinc treatments (control, Si, and NP) (wilcoxon *p* > 0.01), they develop parabolically in the zinc treatments, i.e., an initial strong decrease is followed by a plateau phase and a full recovery towards the end. Compared to the control, photosynthesis proxies were lower in zinc treatments throughout the experiment (wilcoxon *p* < 0.01) with the exceptions of the last sampling (336 h) for the ZnNP treatment and the last two samplings for the Zn treatment.

### Differentially abundant OTUs

**Figure 4:**
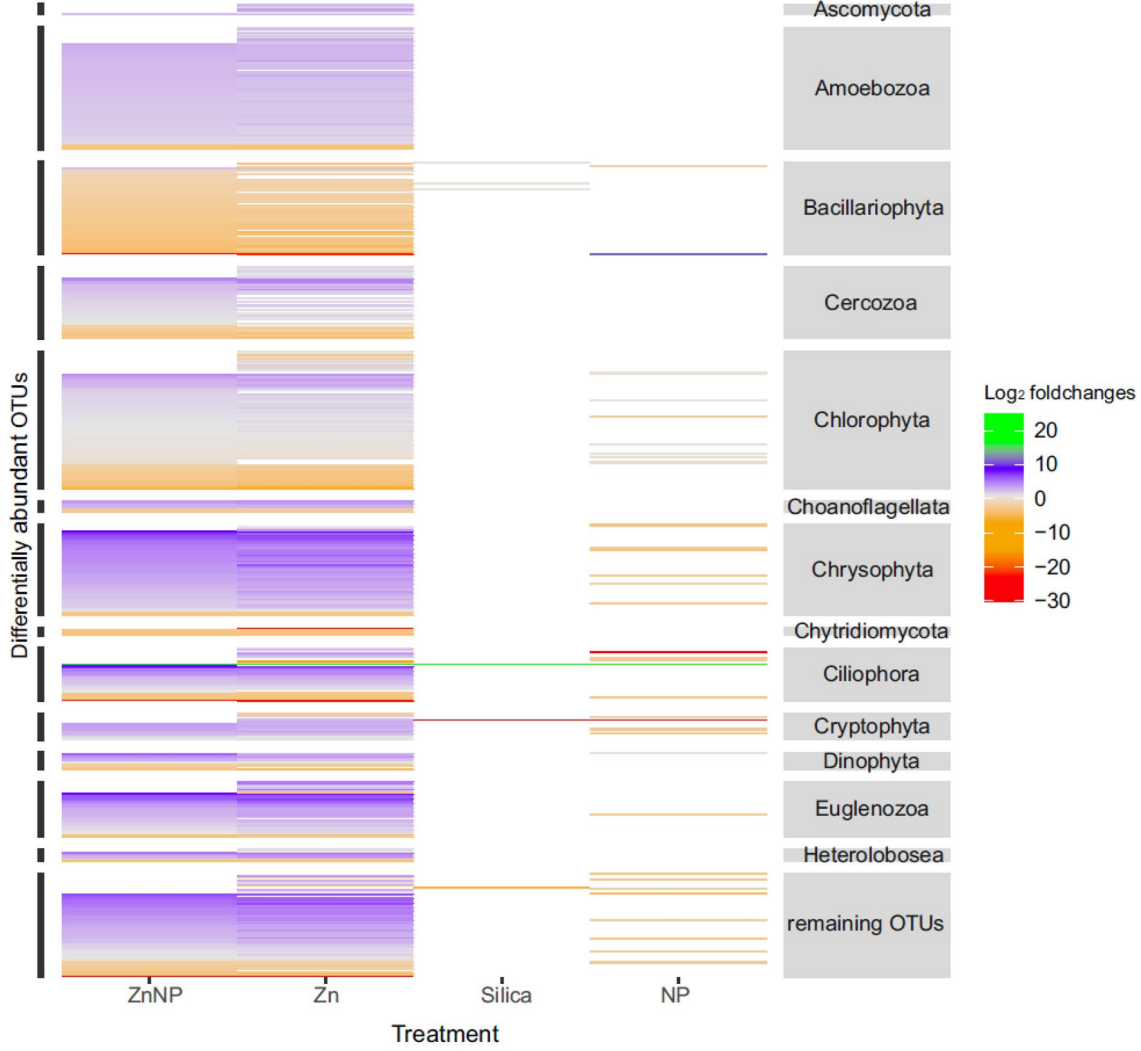
Log_2_-fold changes of differentially abundant OTUs and their affiliation to subgroups compared to the control. Only differences with an adjusted *p* value of 0.01 are shown. Each line represents one OTU. OTUs are sorted in descending order based on their log_2_-fold changes in the ZnNP treatment. Log_2_fold changes are color-coded, the descending order is: green, blue, grey, orange, red. Differences visible here are solely based on the factor treatment.

Community composition shifted differently in treatments and so did individual OTUs. Compared to the control, 519 OTUs (36.81%) were differentially abundant in other treatments (Fig. 4, Table S12) (padj < 0.01). This was most pronounced in the Zn treatment with 351 more abundant and 116 less abundant OTUs and in the ZnNP treatment with 315 more abundant and 118 less abundant OTUs. Patterns between these two treatments were similar as they shared 397 of the differentially abundant OTUs (78.93%). In contrast, in the non-zinc treatments only a minor fraction of OTUs deviated from the control, i.e. 11 OTUs were more abundant and 26 were less abundant and in the NP treatment 4 OTUs were more abundant and 2 were less in the Si treatment.

The differentially developing OTUs again demonstrate the strong effect of zinc and a minor (but present) effect of nanoparticles. Thus, while nanoplastic particles have no effect on the diversity or community composition (Fig. 2) they have an effect on few individual OTUs (accounting for 2.62% of the reads).

In general, the majority of differentially abundant OTUs which were affiliated with Ascomycota, Amoebozoa, Chrysophyta, Cryptomycota and Euglenozoa had a higher abundance as compared to the control with only few exceptions. In contrast, most of the differentially abundant OTUs which were affiliated with Bacillariophyta and Chytridiomycota had lower abundances in the treatments while OTUs affiliated with Cercozoa, Chlorophyta, Choanoflagellata and Dinophyta showed a mixed pattern with a balanced occurrence of some more and some less abundant OTUs.

Some strongly differential abundant OTUs occur with a log_2_fold change of − 30 to 28 (Fig. 4), each with a percentage identity value of less than 98.7%. Therefore reliable affiliations to species are questionable, namely, an unclassified fungi (98.33%), an uncultured Chytriomycota (93.02%) *Holosticha diademata* (93.86%), *Stephanodiscus hantzschii* (98.43%), *Apouronema harbinensis* (98.2%) and *Hemiselmis virescens* (92.59%) (Table S12).

Interestingly, the OTUs most strongly deviating from the control were mostly affiliated with common taxa (Tablr 1). Noteworthy is that *Cryptomonas* sp. seem to react differently to zinc (Zn and ZnNP) as compared to NP.

## Discussion

The environment is subject to many types of pollution, with heavy metals and plastics being of particular importance^93,94^. These pollutants are being released in particular from tires^7,22,34^ and affect microbial communities, especially microeukaryotes^95–97^⁠. Zinc is bound initially to suspended natural particles^98–100^, which explains the deviation from the calculated value (Table S2). Effects on the community structure by zinc are conclusive, as excessive zinc concentrations are toxic^57,101–103^ and lead to lipid peroxidation, decreased chlorophyll content, and reduced activities of carbo-nitrogen and phosphorus acquiring enzymes^59,62^.

The striking impact of zinc after just one hours is especially noteworthy. Zinc entails major cell damages leading to disturbed osmosis, causing the cells to disintegrate in a matter of minutes^104^. Thus the differences between the communities of zinc and non-zinc communities after one hour presumably reflect immediate death and lysis of some individuals following the addition of zinc. This suggests that even short pulses of zinc pollution cause devastating effects, thus, threatening microbial life, ecosystem balance and their function. Further, following this short-term effect is a mid-term effect, as biosorption of metal by microeukaryotes increases with time of exposure leading to enhanced toxicity^60,105–107^.

In general, the community composition changed over time as was to be expected for mesocosm systems^108^ (Fig. 2). However, we observed pronounced differences between treatments indicating significant effects of the tire abrasion substitutes. In particular, community structure was strongly affected by zinc, but not significantly by nanoparticles. In contrast, diversity in terms of OTU richness and effective number of species was barely affected, demonstrating the stability of diversity upon exposure to nanoplastic, zinc and their combination (Figs. S5, S6). In the plastic and silicate nanoparticle treatments we also did not observe strong deviations in the development of the community composition as compared to the control indicating a generally weak effect of nanoparticles. This is in contrast to our initial hypothesis but not necessarily surprising as microeukaryotes are surrounded by a high concentration of particles, including nanoparticles, in their natural environment and microeukaryotic communities should be adapted to varying nanoparticle concentrations^109,110^. In contrast, in the zinc treatments community composition changed over time, i.e. taxon composition changed (Fig. 2) but community structure with respect to species richness and evenness stayed stable. This finding is congruent with discoveries on effects of arsenic, microplastic and nanoplastics in soil^25^.

In all treatments the functional diversity shifted initially towards a stronger contribution of heterotrophic taxa and later back to a more balanced composition, which agrees with the literature regarding functional redundancy^111,112^. As soon as ecosystem functions are disturbed by unfavorable conditions, which lead to the decline of the organisms performing them, the ecosystem strives to compensate by having the functions taken over by other organisms which can cope with the present conditions. Still, this is valid in an open ecosystem with high diversity^111,113^and is more limited in closed mesocosm experiments with limited diversity and dispersion especially regarding the inability of new species to colonize the mesocosms.However, this functional diversity shift was more pronounced in the zinc treatments.

A major finding therefore is that while diversity indices (e.g. effective number of species and evenness) barely reflect an effect of zinc, taxon composition and functional composition do. The functional community composition with respect to trophic modes was strongly imbalanced by the applied tire wear substitutes (Fig. 3). We observed a shift in the functional composition over time in all treatments with a general trend from phototrophic to heterotrophic organisms. The impact of zinc accelerated and amplified this functional shift considerably while there was no corresponding effect for nanoparticles. This was unexpected but conclusive as it is possible that the inert nanoplastic particles formed aggregates and were therefore no longer bioavailable for microeukaryotes^13,114–116^. Alternatively, microorganisms may be used to cope with particles of all size^117–121^. Further the idea that plastics are just a vector for harmful substances could provide an explanation for nanoplastics alone not having a strong effect^2,30,122^. The relative abundance of phototrophs sharply decreased initially in the zinc-treatments causing a short-term increase of mixotrophs and heterotrophs. The relative increase of mixotrophs presumably reflects the abrupt loss of many phototrophic individuals, which were directly affected by the applied substitutes (e.g. cell disintegration)^104,113^as a strong growth of mixotrophs and heterotrophs is unlikely due to the average generation time of around 26 hours^123^. In contrast, despite certain losses of individuals as well, the heterotrophs as a functional group seem to profit early from the zinc addition—most likely due to increased carbon and food availability due to the increased number of dying and dead individuals^46,113^. Within the first days of the experiment, community composition develops towards a community strongly dominated by heterotrophs (≥ 90%). After a couple of days, the detectable increase of heterotrophs is likely based on the decrease of phototrophs and at the same time the actual increase of heterotrophs. Towards the end of the experiment the community composition drifts back towards a more balanced community composition. Thus, the strong initial effect on the phototrophic community may not be explicitly linked to photosynthesis pathways but may be rather taxon-specific and linked to properties, which are not directly related to photosynthesis. A general link to impaired photosynthesis would not allow another organisms group to peform photosysnthesis (i.e. Chlorophyta), but would have a negative effect on all photosynthesis performing organisms.

Based on the initial response, it is not possible to decide whether the decrease in photosynthetic taxa and in community photosynthesis reflects a general effect of zinc on photosynthesis pathways or rather a sharp decrease of the dominating phototrophic taxa (i.e. diatoms), potentially caused by zinc acting on other structures or pathways. Regarding a general effect on phototrophs, photosynthesis and photosynthesis pathways can additionally be directly affect by zinc^60,124^. Further, phototrophic taxa may be more susceptible to zinc for other reasons, i.e. pathways and structures not directly linked to photosynthesis may be affected. For instance, one could argue that while phototrophs have detoxifying mechanisms like osmotic adjustment, excretion of complexing compounds, metal binding compounds, chelation and an antioxidant protection system^60,63,125,126^, heterotrophs may further use particle excretion as an additional way to deal with toxic substances therefore may be slightly better at detoxifying, hence, tolerating zinc^57,127–130^. However, the recovery of phototrophic fraction towards the end of the experiment indicates that there are some photosynthetic taxa capable of dealing with high zinc concentrations. In particular, the dominant algae at the onset of the experiment, i.e. Bacillariophyta, were largely replaced by green algae. This suggests that the observed effect may be limited to or more pronounced in taxa like the Bacillariophyta while other taxa like the (or at least some) green algae rather thrive under high zinc conditions (Fig. S3, S10). This may be linked, for instance, to cell wall properties and cell wall synthesis rather than photosynthesis itself, i.e. silicate cell walls and synthesis pathways may be more strongly impacted by zinc as compared to cell walls made of sugars (cellulose or chitin) in many other taxa. In fact, it is documented that Bacillariophyta are rather sensitive to heavy metals and build deformed frustules, while Archaeplastida, especially Chlorophyta, can better cope with metal pollution^60,63,105,124,130–133^. For instance, the chlorophyte species *Halochlorella rubescens* thrived well in the zinc treatments. The high abundance is coherent with the literature, since this species was used in the wastewater sector^134^ and was investigated especially for zinc removal from wastewater^135^.

The relative abundance of heterotrophic and mixotrophic fractions increased initially. One could assume that the mixotrophs took over the photosynthetic ecosystem function during this period until the obligate phototrophs eventually recovered. The photosynthesis proxies contraindicate this, i.e. the Fv/Fm values are collapsing (Fig. S7). Thus, mixotrophs probably rely largely on their ability to feed heterotrophically during this period.

This observed switch to a predominantly heterotrophic community is reasonable assuming that the phototrophs in the original community are vulnerable to zinc and that heterotrophs profit from increased food availability in form of dead algal biomass, i.e. it is likely that especially photosynthetic organisms serve as food source^43,52,56,59^ (Fig. S7). The assumed selective advantage of heterotrophs during the initial phase of the experiment is consistent with a decreasing importance of mixotrophs and a further increasing importance of obligate heterotrophs.

The addition of zinc also resulted in a lower relative abundance of parasites (Fig. S9). As most parasites are host-specific, the relative abundance of parasite and host is likely coupled^136–138^. As zinc is causing a decline in phototrophs, it will directly (e.g. disturbed osmosis) or indirectly (e.g. killing the host) affect parasite relative abundance^139^. The decrease of parasites thus suggests that many taxa were parasites of diatoms^138,140,141^⁠.

We did not observe a strong effect on fungi (Fig. S9) and fungal diversity is not affected by zinc, suggesting that a direct impact is unlikely^142^. This is not surprising as the fungal fraction is low in pelagic freshwaters^143,144^ and the nature of compositional data may obscure shifts especially in rare taxa. In summary, it is evident that zinc is strongly unsettling the balance between heterotrophs and phototrophs.

In contrast to our expectations, nanoparticles did not strongly affect microeukaryotic communities or functional composition. We observed no combined effect of zinc and nanoplastic. Even though we have to reject our hypothesis of direct effects on the community and functional level, this finding is plausible: Microeukaryotes aresurrounded by a high concentration of particles with different surface properties (e.g. clay, silt, bacteria). Thus, they are likely well adapted to cope with them^117–121^. Further, nanoparticles cluster easily to hetero- and homoaggregates^13,114–116^ and are therefore less likely to have direct interactions with microeukaryotes. Still, plastic leachates and surfactants are known to negatively affect microorganisms in various ways^145,146^ but rather due to the respective surfactants or leachates than the nanoparticles themselves^29,147,148^. Our results on tire wear substitutes back this idea as zinc (being a leachate from tire wear) has a strong impact while nanoplastic (as potential vector) is rather neutral (Fig. 2, 3, S7). It should also to be noted that nanoplastic remains in the environment for centuries^18^, thus a short- to medium-term exposure likely does not reveal all effects. However, with an average generation time of 26 h the investigated runtime allows to observe effects over multiple generations^123^. Furthermore, it should be noted that nanoplastics normally occur in combination with microplastics, through this combination the effects in natural environment can deviate^113^. With the given half-life of tires, which are made to persist, it becomes obvious that the pollution caused by tires is increasing. With increasing pollution from tires, the chances are rising that at some point microorganisms develop ways to utilize and break down their components, thus playing an important role in biodegradation and bioremediation^149–151^. However, this should not be relied upon, because even if this is the case the pollution level will be so advanced that damage caused to biodiversity is already irreversible.

### Differential abundant OTUs

On the level of individual OTUs zinc caused large scale community modulations (pairwise adonis *p* < 0.01, DESeq2 Wald test *p* < 0.01) (Fig. 2, 3, 4), while effects of nanoparticles were less prominent. In particular, a difference between polystyrene and silica nanoparticles became apparent, which was only detectable on this level. Corresponding to the community and functional group level effects most phototrophic taxa decreased after exposure to zinc. This comprises species affiliated with e.g. the chrysophyte genera *Mallomonas* and *Synura*^152,153^⁠, the green algal genus *Micractinium*^154^ as well as many diatom genera (e.g. *Sellaphora*, *Fistulifera* and *Nitzschia)*^131,132,155^. While the energy providing photosynthesis as well as cellular integrity is impaired, the degree of impairment is not uniform among taxa^43,52,56,59,104^. As an exception, the diatom *Conticribra weissflogiopsis,* increased in relative abundance and may be an interesting candidate for restoration and treatment of zinc polluted waters. Again in concert with the community and functional group level findings OTUs affiliated with preferably heterotrophic taxa such as Amoebozoa, (heterotrophic) Chrysophyta and Euglenozoa were generally increasing after zinc exposure^96^, which is conclusive as they benefit from impaired organisms (e.g. *Spumella*, *Poteriospumella*, *Pedospumella*, *Chlamydomonas*)^156–158^ and several of these species are rather metal insensitive (e.g. *Vannella simplex*, *Bodo saltans*, *Rhynchomonas nasuta)*^159–161^. While zinc affected a broad spectrum of OTUs, nanoparticles affected only few. Silica nanoparticles lead to only a few differentially abundant OTU (e.g. *Cyclotella meneghiniana*), while polystyrene nanoparticles lead to several differentially abundant OTUs. The weak effect in particular of silica nanoparticles is plausible as silica particles (e.g. clay minerals) occur naturally in the environment and protists presumably are adapted to dealing with such particles.

Nanoplastic may not cause large scale community modulations, but individual species can be affected as already shown for bacterial phytoplankton^131,162^. Interestingly, a number of OTUs (e.g. affiliated with *Cryptomonas* sp., *Acrispumella msimbaziensis*, *Neobodo designis*, *Spumella* sp., *Chlorella* sp.) (Tables 1, S12) which increased in relative abundance in the zinc-containing treatments were decreasing when exposed just to NP. It is likely that drawbacks of nanoplastic become rather visible on higher trophic levels as nanoplastic accumulated due to feeding on microeukaryotes^30,116,163,164^.

**Table 1:**
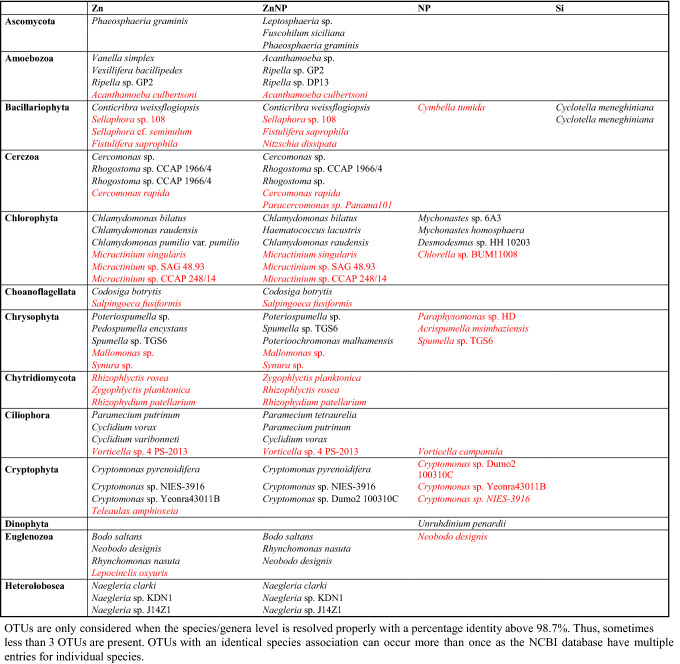
Showing the top 3 increased (black) and top 3 decreased (red) differential abundant OTUs for individual groups and treatments.

However, the individual groups show generally a clear pattern with increasing (e.g. Amoebozoa, Chrysophyta) or decreasing (e.g. Bacillariophyta) abundances with individual exception due zinc exposure. While different patterns for the remaining, Chlorophyta, Dinophyta, Cercozoa, Choanoflagellata and Heterolobosea, are present. The relative amount of increasing and decreasing OTUs isbalanced implying unclear patterns for these groups. Which is conclusive as microdiversity is contrasting among certain phyla or groups and is therefore not a trait specific for certain phyla or groups, therefore, tolerances and deficiencies are different even for closely related taxa. Individual OTUs show different reactions while being within the same genus (e.g. *Acanthamoeba*, *Cercomonas*)^96,165,166^. After all, when working with compositional data one has to note that increasing abundances can have three reasons: (i) the abundance is actually increasing, (ii) the abundance of other groups are decreasing, thus, an ostensible increase is visible or (iii), a mixture of both. This peculiarity may lead to a distorted perception and masking of effects.

The effects of tire abrasion as derived from tire abrasion substitutes, however, may behave different in the environment, as tire wear abrasion is a non-point source of pollution that tends to occur in pulses, in particular after long dry period followed by heavy rainfall^22,167–172^. It is therefore likely that immediate effects (e.g. disintegration of cells) are more important than mid- to long-term effects for pelagial organisms^104^ and more pronounced than indicated by our study. Finally, the used tire abrasion substitutes presumably provide a simplified sight, as tires consist of far more potential hazardous substances^170,173–175^. Due to the quantitative importance of tire abrasion and the here and elsewhere already documented^176,177^ significance of different tire abrasion substitutes the impact of tire abrasion on the surrounding environments is drastic with unpredictable long term consequences requiring further examination.

## Conclusion

Ongoing pollution by tire abrasion contaminants, like nanoplastic and zinc, will accumulate, reach high concentrations in the environment, and trigger effects in biological communities^30,116^—it is just a matter of time. Here we demonstrated the significance of tire abrasion substitues, in particular zinc, for eukaryotic microbial communities with a pronounced short-term functional imbalance and lasting effects on species composition. Tire abrasion strongly affects microeukaryotic communities, but predominantly due to the release of leachates, while the tire wear particles have a minor effect. Zinc has an immediate effect on susceptible cells (death, photosynthesis) and a medium-term effect on the community structure and functional diversity. However, the functional diversity is restored over time and general community diversity is not affected at any point. Still, long-term effects may occur causing unpredictable harm to protists especially if nanoparticles contain surfactants or release leachates.

It would be interesting to investigate how the microeukaryotic community behaves after stressor release, in particular after zinc exposure, and if the potential to shift back to the initial community profile is still present or if it is irreversible lost due to stressor exposures. In future studies methods such as RNA based analyses (metatranscriptomics), mass spectrometry (proteomics, metabolomics) and ecophysiological methods may provide more insights into the functional basis of the community response.

## Supplementary Information

Supplementary Information 1.

Supplementary Information 2.
