# Supplementary material for: Microbial community shifts induced by plastic and zinc as substitutes of tire abrasion
Source: Sci Rep. 2022 Nov 4;12:18684. doi: 10.1038/s41598-022-22906-6 (PMC9636222; doi:10.1038/s41598-022-22906-6)
Supplement: Supplementary file 1 — Supplementary Information 1. [file 41598_2022_22906_MOESM1_ESM.docx]

| **Supplementary figures:** |
| --- |
| **Microbial Community Shifts Induced by Plastid and Zinc as Substitutes of Tire Abrasion** |
| **G. Sieber^1*^, D. Beisser^1, 2^, J. Olefeld^1^, M. Shah^1^, M. Schumann^3^, B. Sures^2,3^ & J. Boenigk^1,2^** |
| **^1^ Biodiversity, University of Duisburg-Essen, Essen, Germany** |
| **^2^ Centre for Water and Environmental Research, University of Duisburg-Essen, Essen, Germany** |
| **^3^ Aquatic Ecology, University of Duisburg-Essen, Essen, Germany** |
| ***Corresponding author: Guido Sieber (guido.sieber@uni-due.de)*** |


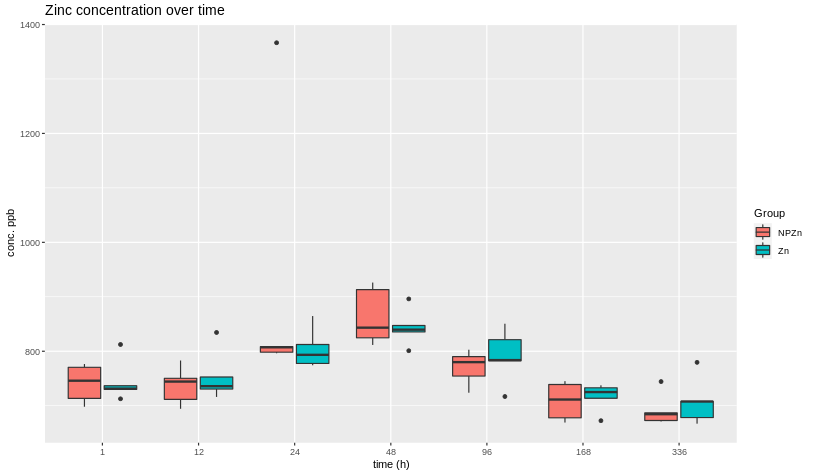


Figure S1: Zinc concentration in ppb (parts per billion) over time for the ZnNP and Zn treatment


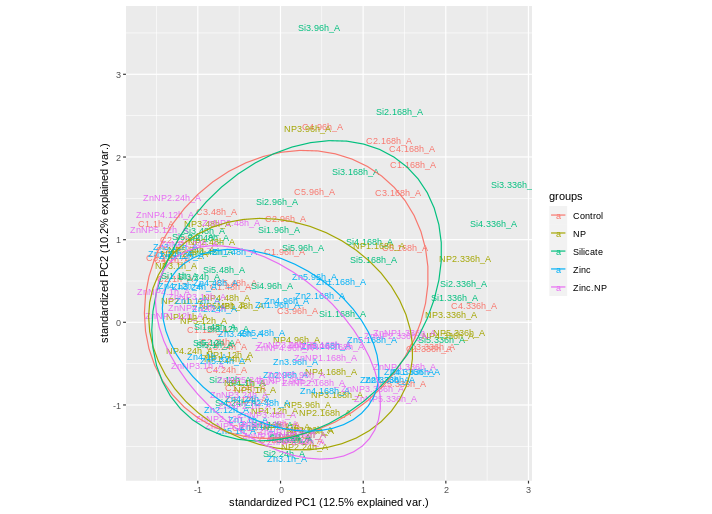


Figure S2. PCoA calculated with ASVs obtained from dada2


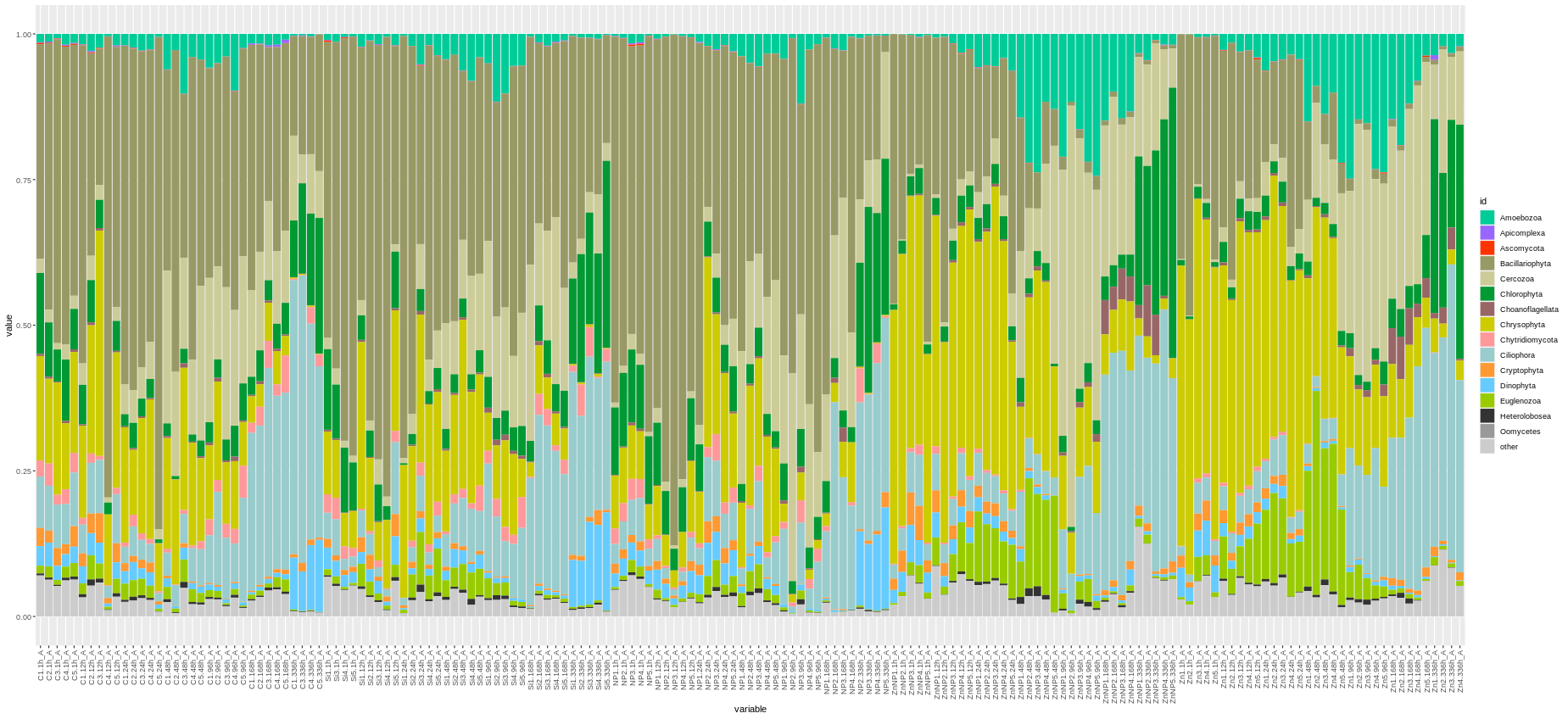


Fig. S3: Showing relative communtiy compositions of treatments over time based of higher level groups


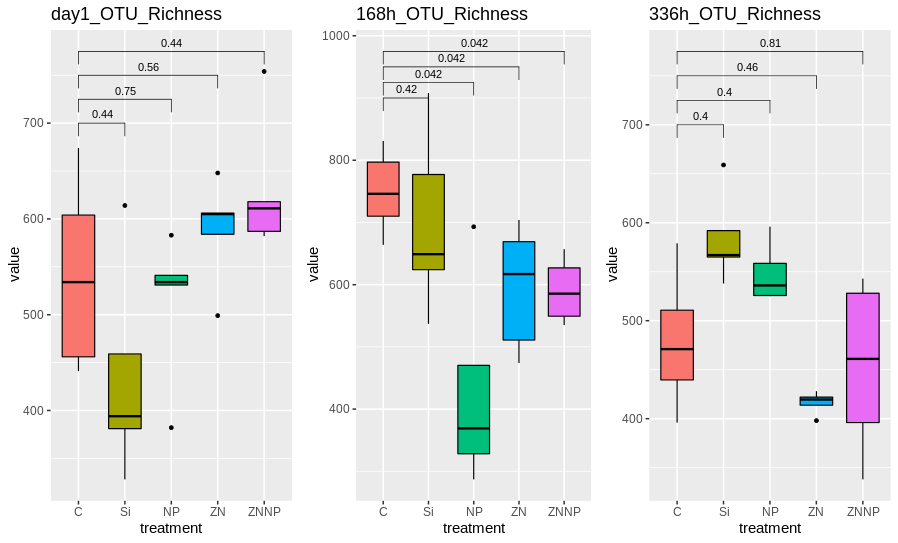


Figure S4: Boxplot depicting OTU richness. Braces show adjusted p-value . Treatments are color-coded. The first sampling day is merged (1h, 12h, 24h), thus portraying the first day.


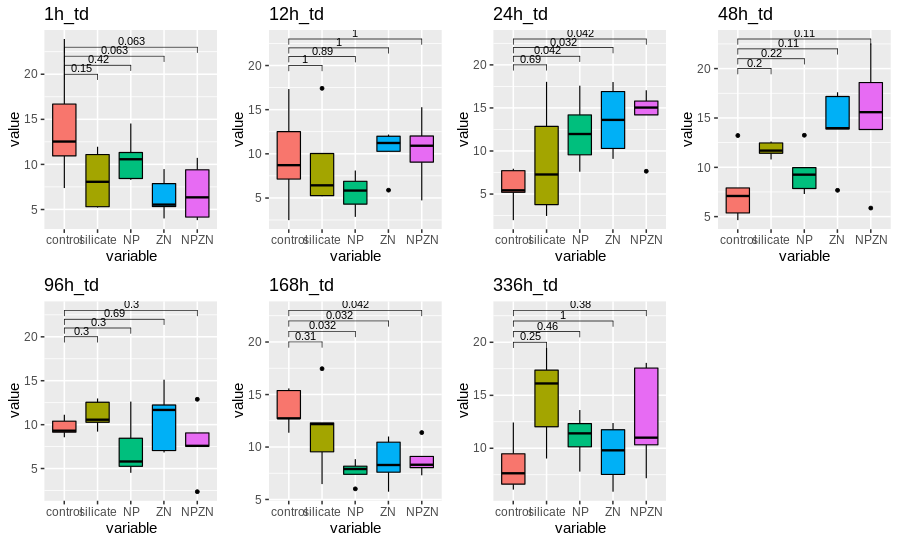


Figure S5: Boxplot of true diversities of the treatments. Braces show p values of the true diversity compared with the control. Treatments are color coded.


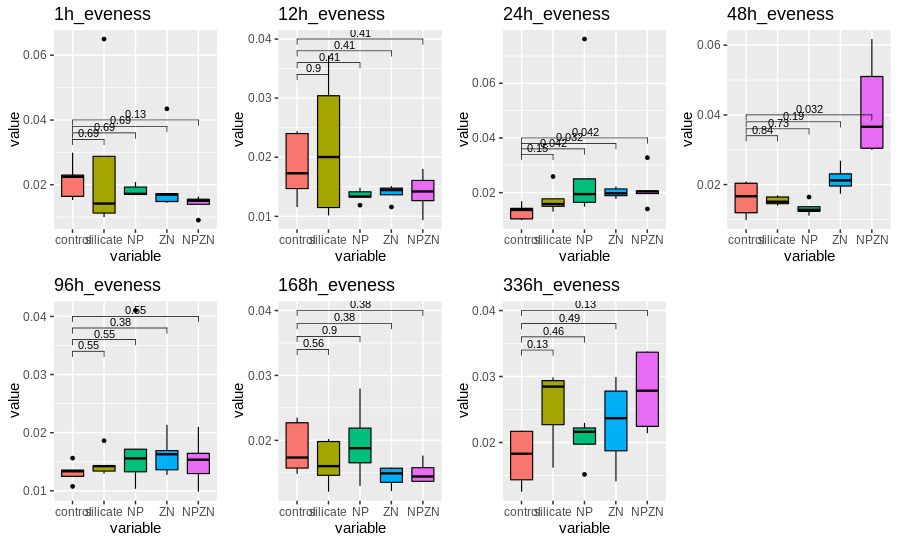


Figure S6: Evenness boxplots of the treatments. Braces show p-values of comparisons with the control. Treatments are color-coded.


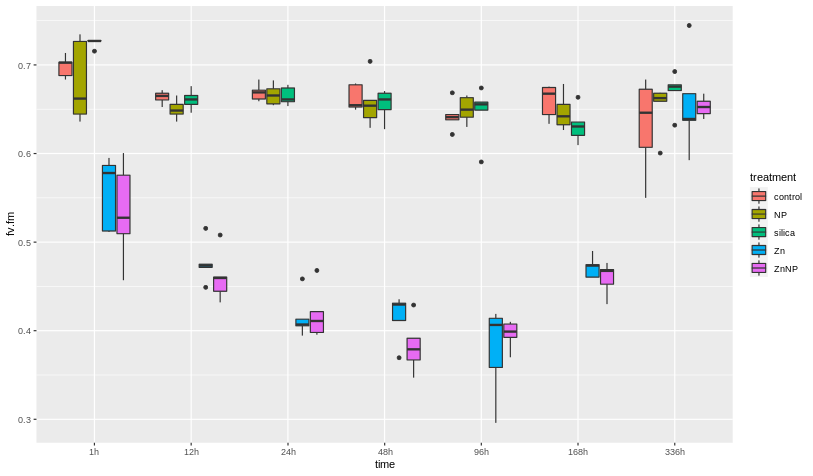


Figure S7: Fv/Fm values of the treatments over time.


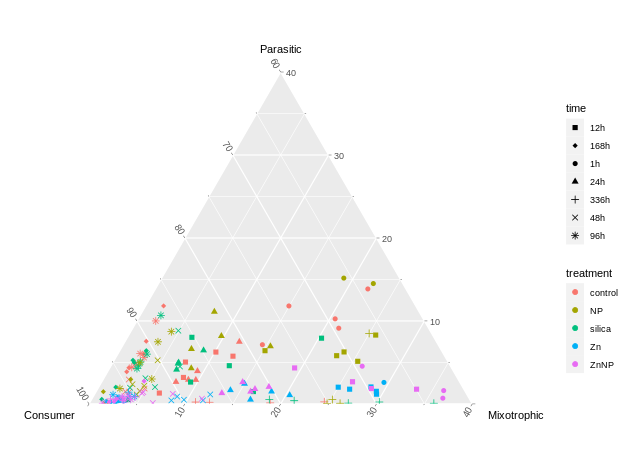


Figure S8 Ternary plot : Showing differences in consumer, parasitic and mixotrophic relative abundance. Based on rarefied data. For better visualization the three groups were considered as 100%


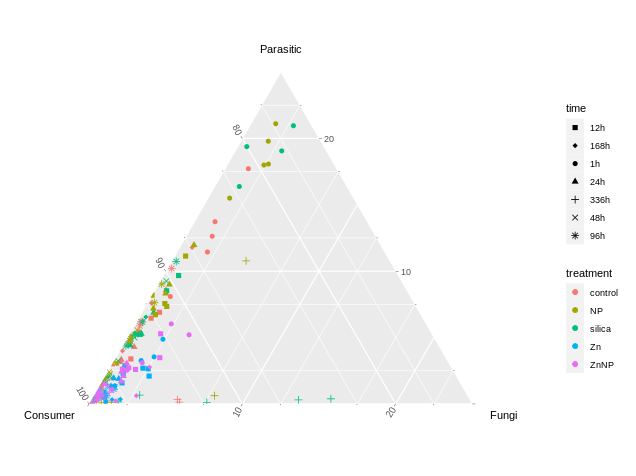


Figure S9: Showing differences in consumer, parasitic and fungal relative abundance. Based on rarefied data. For better visualization the three groups were considered as 100%


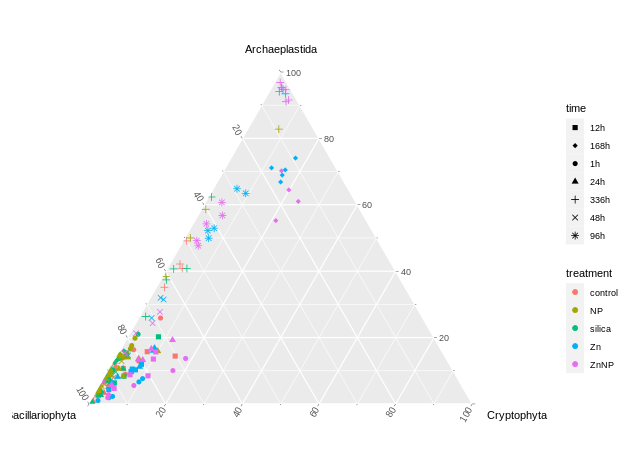


Figure S10: Showing differences in Archaplastida, Bacillariophyta and Cryptophyta relative abundance. Based on rarefied data. For better visualization the three groups were considered as 100%
